# The Broad Host Range Phage vB_CpeS_BG3P Is Able to Inhibit *Clostridium perfringens* Growth

**DOI:** 10.3390/v14040676

**Published:** 2022-03-25

**Authors:** Sisi Huang, Yuan Tian, Yongjuan Wang, Pilar García, Banhong Liu, Rui Lu, Liting Wu, Hongduo Bao, Maoda Pang, Yan Zhou, Ran Wang, Hui Zhang

**Affiliations:** 1 College of Veterinary Medicine, Nanjing Agricultural University, Nanjing 210095, China; 13219030916@163.com; 2 Jiangsu Key Laboratory for Food Quality and Safety-State Key Laboratory Cultivation Base of MOST, Jiangsu Academy of Agricultural Sciences, Nanjing 210014, China; ann1491986585@163.com (Y.T.); liubanhong9821@163.com (B.L.); rainbowmaker225@163.com (R.L.); zh851200@yahoo.com (L.W.); baohongduo@163.com (H.B.); 20150027@jaas.ac.cn (M.P.); zhou.yan.77@hotmail.com (Y.Z.); w1206186910@163.com (R.W.); 3 School of Food and Biological Engineering, Jiangsu University, Zhenjiang 212013, China; 4 Jiangsu Agri-animal Husbandry Vocational College, Taizhou 225300, China; yongjuanwang@jsahvc.edu.cn; 5 Dairy Research Institute of Asturias (IPLA-CSIC), 33300 Villaviciosa, Asturias, Spain; pgarcia@ipla.csic.es; 6 School of Food and Biological Engineering, Shaanxi University of Science and Technology, Xi’an 710021, China

**Keywords:** *Clostridium perfringens*, phage, antimicrobial agent, poultry industry

## Abstract

*Clostridium perfringens* is an important pathogen for both humans and animals, causing human foodborne disease and necrotic enteritis in poultry. In the present study, a *C. perfringens*-specific phage, vB_CpeS_BG3P (designated as BG3P hereafter), was isolated from chicken farm sewage. Both electron microscopy and phylogenetic analysis suggested that phage BG3P is a novel phage belonging to *Siphoviridae* family. Phage BG3P exhibited a broad host range against different *C. perfringens* isolates (90.63% of strains were infected). Sequencing of the complete genome revealed a linear double-stranded DNA (43,528 bp) with 28.65% GC content. After sequence analysis, 73 open reading frames (*orf*s) were predicted, of which only 13 were annotated with known functions. No tRNA and virulence encoding genes were detected. It should be noted that the protein of *orf 15* has 97.92% homology to *C. perfringens*-specific chloramphenicol resistance protein, which has not been reported for any *C. perfringens* phage. Phylogenetic analysis of the ssDNA binding protein demonstrated that this phage is closely related to *C. perfringens* phages phiSM101 and phi3626. In considering future use as an antimicrobial agent, some biological characteristics were observed, such as a good pH (3–11) stability and moderate temperature tolerance (<60 °C). Moreover, bacteriophage BG3P showed a good antimicrobial effect against *C. perfringens* liquid cultures. Thus, phage treatment with MOI ≥ 100 completely inhibited bacterial growth compared to untreated cultures. Although phage BG3P shows good lytic efficiency and broad host range in vitro, future development and application may need to consider removal of the chloramphenicol-like resistance gene or exploring its lysin for future antibacterial applications.

## 1. Introduction

*Clostridium perfringens* is a Gram-positive, strictly anaerobic, spore-forming bacterium that is widely distributed in nature [1]. Spores of the pathogen can persist in soil, feces or the environment, and the bacterium causes many severe infections in humans and animals, including food poisoning, gas gangrene, and non-foodborne gastrointestinal infections in humans, and necrotizing enteritis in animals [2]. *C. perfringens* is classified into one of five types (A, B, C, D, or E) based on the toxin production. All of these produce alpha-toxin (phospholipase C, lecithinase), which plays a major role in the pathogenesis of gas gangrene. *C. perfringens* type C also produces beta-toxin, which is implicated in enteritis necrotic, and enterotoxin of *C. perfringens* type A is involved in food poisoning. Thus, once the bacterial population reaches a certain density (>10^4^ CFU/g), toxin production is triggered, which induces *C. perfringens* type A food poisoning, considered the most common bacterial food poisoning in industrialized countries [3].

In chickens, *C. perfringens* is the major pathogen of necrotizing enteritis, and its acute form can lead to increased mortality in broiler flocks. In addition, due to the intestinal mucosal damage caused by this bacterium, the subclinical form can lead to reduced digestion and weight gain. Although *C. perfringens* can be controlled with antibiotics, there is an increasing pressure to ban the use of growth-promoting antibacterial agents in poultry. In the European Union (EU), antimicrobial growth promoters (AGPs) were banned from animal feeds in 2006 (Regulation 1831/2003/EC) because of concerns about the increasing prevalence of antibiotic resistance among clinical bacteria [4,5]. Removal of these antimicrobials will dictate the need for alternative compounds, in order to achieve the same high level of food-animal production achieved with AGPs. Indeed, the reduced or banned use of antibiotics in some countries has already resulted in increased cases of necrotic enteritis in poultry [6]. In China, antibiotic feed additives for animals were withdrawn in 2020; therefore, the infection rate in animals is expected to continue increasing.

Bacteriophages (or phages) are a group of viruses widely distributed in nature and whose life cycle is strictly associated with the bacterial cell. Indeed, the propagation of phages inside their host (lytic cycle) has as a consequence the death of the cell; therefore, they can be used as effective alternatives to antibiotics for pathogen control [7]. Positive results of the use of bacteriophages in fighting bacterial infections have contributed to the development of phage therapy for the treatment of diseases in both human and animals [8,9]. With the continuous increase in antibiotic-resistant pathogens, there has been a resurgent interest in phage biology and in the use of phages, or phage gene products, as antibacterial agents [10,11] for veterinary and human medicine, as well as in the bioindustry worldwide.

Previous studies have focused on developing phages or their lysins against *C. perfringens* strains closely related to necrotic enteritis in broilers [12,13]. Miller et al. [12] developed a phage cocktail (INT-401), composed of five phages, that was orally and by spray administered to *C. perfringens-*challenged broiler chickens, reducing mortality rates and improving the body weight gain and feed-conversion ratio. More recently, phage φCJ22 was used as a dietary supplement in experimental necrosis-affected broiler chickens. The results showed decreased lesions and mortality rates, although body weight gain and feed efficiency were significantly lower in phage-fed groups than that in untreated animals [9]. In addition to phages, phage-derived proteins (endolysins) have been explored as antimicrobials against this bacterium [14,15,16]; however, the main drawback is the delivery of these proteins to animals, and therefore no successful in vivo assays have been published to date using phage lytic proteins [17].

Because of the phage specificity, a wide collection of phages is essential to ensure the infection of as many strains as possible. Here, we have isolated and characterized a new phage-infecting *C. perfringens* strain of farm origin. The complete genomic characterization and biological properties point towards a wide host range phage. In this study, we will analyze the related lytic effects of this phage in order to provide effective strategies for the control of *C. perfringens* in the poultry industry.

## 2. Materials and Methods

### 2.1. Strains and Culture Conditions

The *C. perfringens* strains used in this study are shown in Table 1, including *C. perfringens* collection strains (ATCC 13124, CMCC 64722, CMCC 64723, and CMCC 64724) and 32 *C. perfringens* isolates from chicken feces, liver, intestinal, spleen, and heart samples collected in Shandong Province, China. The toxin typing method of the 32 *C. perfringens* strains was carried out with reference to the method and designed primers of van Asten et al. [18]. Briefly, DNA extraction was performed using the boiling method, and multiplex PCR was used to identify the toxin typing of the strain. Only α toxin is type A; α, ε, β toxin is Type B; α, β toxins is Type C; α, ε toxins is Type D; α, ι toxins is Type E. ATCC 13,124 was used as a positive control. PCR was performed with an initial denaturation at 95 °C for 5 min, followed by 35 cycles of 95 °C for 30 s, 57 °C for 30 s, and 72 °C for 30 s. Amplification products were detected by gel electrophoresis. The primer sequences are shown in the Table 2. All *C. perfringens* strains were used as the indicator strain for isolation of phages using sewage suspensions from chicken farms. All *C. perfringens* strains were routinely grown anaerobically at 37 °C in Anaerobic Meat Liver (AML) Broth (Qingdao Hope Bio-Technology Co., Ltd., Qingdao, China), using the LAI-3 Anaerobic incubator (Shanghai Longyue Instrument Equipment Co., Ltd., China). All strain stocks were stored at −80 °C in Brain Heart Infusion (BHI) Broth (Qingdao Hope Bio-Technology Co., Ltd., Qingdao, China) supplemented with 30% glycerol. *Staphylococcus aureus*, *Salmonella* enteritidis, *E. coli,* and *Listeria monocytogenes* were grown in BHI medium at 37 °C. For the double-layer assay (see below), TY medium (OXOID Co., Ltd., Basingstoke, UK) was used.

### 2.2. Isolation and Purification of Bacteriophages

Isolation of phages was performed following the previously described method for *C. perfringens* phage enrichment from sewage suspensions [19]. Briefly, the 36 *C. perfringens* cultures were mixed with the centrifugally filtered sewage supernatant for 24–48 h and centrifuged at 10,000× *g* at 4 °C for 30 min. Then, the supernatant was filtered with a 0.22 μM to obtain enriched water samples. The double agar overlay method was then used to detect the presence of phages in the enriched water sample. A total of 0.5 mL host bacteria was mixed with 4 mL TY molten soft agar, which was distributed evenly to solidify on a TY standard agar plate. Aliquots (10 μL) of the enriched water sample were dropped on soft agar, and anaerobic culture was conducted overnight at 37 °C. Clear plaques will appear where the soft agar is dripped with enriched water samples containing phages. Lysis plaques were suspended into SM buffer (50 mM Tris-Cl, 100 mM NaCl, 8 mM MgSO_4_, pH 7.5), centrifuged, filtered, and stored as “spot filtrate” at 4 °C. Dilutions of spot filtrate were mixed with host bacteria in a molten TY soft agar, which was poured on a TY plate and incubated. Isolated plaques were suspended again into SM buffer to purify the phage. Purified phages were obtained after 3–5 rounds of purification. This purified phage was used to prepare high titer (10^8^–10^9^ PFU/mL) phage stocks for further analyses.

### 2.3. Preparation of Phages for Transmission Electron Microscopy

High titer stocks were prepared in SM buffer by PEG-8000 precipitation [20]. The pellet was rinsed three times in 0.1 M ammonium acetate, the supernatant discarded, and the pellet gently resuspended in 0.5 mL distilled water, which was then filtered (0.22 μM) and stored under refrigeration until required. A drop (10 μL) of phage concentrate was applied to a 200-mesh grid and left for 1 min, excess was liquid drawn off with filter paper, and the grid was allowed to air dry. A drop of buffered 1%-phosphotungstic acid (aqueous, pH 6.5) was applied, blotted off immediately, and the grid was allowed to air dry. The grid was then loaded into a Hitachi H-7650 transmission electron microscope (Hitachi, Japan), and phages were examined at 300,000 magnification.

### 2.4. One-Step Growth Curve

The one-step growth curve was obtained using the improved method described by Hyman et al. [21]. Briefly, host strains were cultured to the logarithmic growth stage, and then cultures were infected with the phage at a multiplicity of infection (MOI) of 10. Infected host bacteria were incubated at 37 °C for 10 min and then centrifuged to remove culture medium and unadsorbed phages. After washing the pellet twice, preheated AML broth was added to resuspend the cells. Cultures were incubated with shaking at 37 °C, and samples were taken every 10 min for a total of 2 h. The double-layer method was used to determine the phage titer. All assays were performed in duplicate.

### 2.5. Host Range Analysis

The host range of the phage was identified by testing the lytic ability in 32 *C. perfringens* strains, isolated from different regions in China by National Standard of China GB/T 26425-2010. The isolates were inoculated in TY agar by the double agar overlay assay. Briefly, cultures of each strain (0.5 mL) were mixed with 4 mL TY molten soft agar and distributed on a TY standard agar plate. After the top agar was solidified, purified phage (10 μL) was dropped on the top agar. After 20 h of incubation at 37 °C, clear zones were recorded to reflect the inhibition of bacterial growth. Each experiment was repeated three times.

### 2.6. Bacterial Challenge Assays

Multiplicity of infection was determined by reference to the improved method [22]. Host bacteria cultures were obtained by inoculating a single colony in the AML medium and incubated until the logarithmic growth phase (OD = 0.6, 2 × 10^7^ CFU/mL) was reached. Aliquots (2 mL) of these cultures were mixed with the phage and incubated at 37 °C for 3.5 h. Multiplicity of infection (MOI) was calculated to be 1000, 100, 10, 1, 0.1, 0.01, and 0.001. Cultures were centrifuged at 8000 rpm for 30 min, and the supernatant was used to determinate the phage titer.

### 2.7. Thermal and pH Stability of the Phages

For determining thermal stability, phage samples in SM buffer were incubated at 30 °C, 40 °C, 50 °C, 60 °C, 70 °C, and 80 °C. Aliquots were taken after 30 min and 60 min to determine the phage titer by the double-layer agar method. For analysis of pH stability, samples of the phage were mixed in a series of tubes containing SM of different pH values (2~13, adjusted using NaOH or HCl), incubated for 2 h at 37 °C, and then titered by the double-layer agar method.

### 2.8. Phage Adsorption and Lytic Assays

Phage adsorption was performed as previously described [23]. Cells of *C. perfringens* BG3 (2 × 10^7^ CFU/mL) were infected with the phage suspension (2 × 10^5^ PFU/mL) to give an MOI of 0.01 and incubated at 37 °C. Aliquots (100 μL) were taken at specific time points of 5, 10, 15, 20, 25, and 30 min and immediately diluted in 0.9 mL of cold AML broth. Following centrifugation (12,000× *g*, 5 min), the supernatants containing un-adsorbed phages were diluted and titered by the double-layered agar assay. Adsorption was expressed as percent decrease of phage titer in the supernatant. 

For the lytic activity of the phage, fresh *C. perfringens* cultures (OD_600_ 0.6, 2 × 10^7^ CFU/mL) were mixed with the phage to reach an MOI of 100. Infected cultures were incubated statically at 37 °C, and OD_600_ was measured every 30 min to reflect the growth of *C. perfringens* for a total of 6 h. Bacterial growth without the addition of phage was also determined for a period of 6 h. Each experiment was repeated three times, and the average data with standard deviation were presented.

In order to determine the lytic efficiency, we studied the lysis effect of the phage at different concentrations. Cells of *C. perfringens* BG3 (2 × 10^7^ CFU/mL, 1 mL) were infected with diluted phage suspension (1 mL) to give an MOI of 1000, 100, 10, 1, 0.1, 0.01, and 0.001 and incubated at 37 °C. The OD_600_ was tested every 30 min and lasted for 6 h.

### 2.9. Phage Genome Sequencing and Bioinformatics Analysis

The genomic DNA of the phage was extracted using the Norgen Biotek Phage DNA Isolation Kit (Norgen Biotek Corp. Thorold, ON, Canada). Briefly, phage genomic DNA was obtained through 4 steps of propagating the phage in liquid cultures and preparing the phage supernatant containing 10^9^ PFU/mL, lysate preparation, sample binding to column, column washing, and DNA elution. Genomic DNA sequencing was performed using the Illumina Novaseq platform (Shanghai Personalbio Technology Co., Ltd. Shanghai, China). The standard Illumina TruSeq Nano DNA LT library preparations (Illumina TruSeq DNA Sample Preparation Guide) were used to construct the required online genome libraries. Genemarks [24] software was used to predict the protein-coding genes of the genome, and the sequence alignment of the coding genes was completed by Diamond software [25]. The putative protein function of the ORFs was annotated using the NR database. Diamond BLASTp was used to compare the protein sequences encoded by genes with the protein sequences in the database, to identify the function of the presumed proteins. Non-coding RNAs in the genome were predicted by comparing the ncRNA annotation database http://rfam.xfam.org/ (accessed on 13 August 2021) [26]. Phylogenetic analysis was conducted using MEGA (version X _10.2.6, Auckland, New Zealand).

### 2.10. Determination of Gene Transfer from Phage to Host Strain

Gene transfer between phage and host bacteria was focused on bacteriophage *orf 15*, as the protein encoded by this *orf* was determined to be 97.92% similar to the chloramphenicol resistance protein of *C. perfringens* (WP_111946556.1). The primers were designed by *orf 15*, and the 32 clinical strains of *C. perfringens* were tested by PCR to observe whether they carried the *orf 15* encoding gene.

In order to explore whether the chloramphenicol-like resistance gene *orf 15* (Cm^R^) would be transferred into host strain, phages were mixed (10^5^ PFU/mL) with Cm^R^ negative *C. perfringens* strains (ATCC 13124, CMCC 64722, BG5, RZ-5, BG1, BG3, BC8, BX5, BG4, and BX9-1) (10^7^ CFU/mL) and incubated at 37 °C for 72 h. In addition, the mixture was streaked onto TSC plates with or without chloramphenicol (10 μg/mL) to further determine whether there was colony growth resistant to chloramphenicol. If single colonies could be observed, the DNA of the resistant host strain was amplified by PCR with primers of *orf 5*, *orf 11*, *orf 25*, and *orf 28*. The primers sets used are listed in Table 3. Samples of this mixture (1 mL) were collected at 12 h, 24 h, 48 h, and 72 h and washed with PBS 3 times by centrifugation. Total RNA of these centrifuged host strains were extracted, and the expression of the *orf 15* was detected by RT-qPCR. In brief, total RNA was extracted from the mixture using Trizol (Vazyme Biotech, Nanjing, China) according to manufacturer’s instructions. Reverse transcription was carried out using a Reverse Transcription Kit (Vazyme Biotech) according to the manufacturer′s instructions. Quantitative real-time PCR was used to evaluate the transcription of *orf 15*. Quantitative PCR was performed using 5 μL of cDNA, 10 mM primer, and 10 μL of CHAMQ SYBR qPCR Master Mix (Vazyme Biotech, Nanjing, China) in a final volume of 20 μL per reaction. The cycling parameters for quantitative PCR were as follows: pre-denaturation step was 95 ℃ for 30 s, followed by 40 cycles of 95 °C for 10 s, and 60 °C for 60 s. The dissolution curves were 95 ℃ for 10 s, 60 ℃ for 60 s, and 95 ℃ for 15 s. All samples were characterized by a corresponding cycle threshold (Ct) value. Phage RNA was used as the standard curve template. Negative samples gave no Ct value.

### 2.11. Statistical Analysis

Statistical analyses were performed with SPSS software (IBM Corporation, Armonk, NY, USA). Comparisons were analyzed using nonparametric one-way analysis of variance (ANOVA) with Bonferroni’s multiple-comparison post-test.

## 3. Results

### 3.1. Isolation and Characterization of C. perfringens Phage

**Figure 1:**
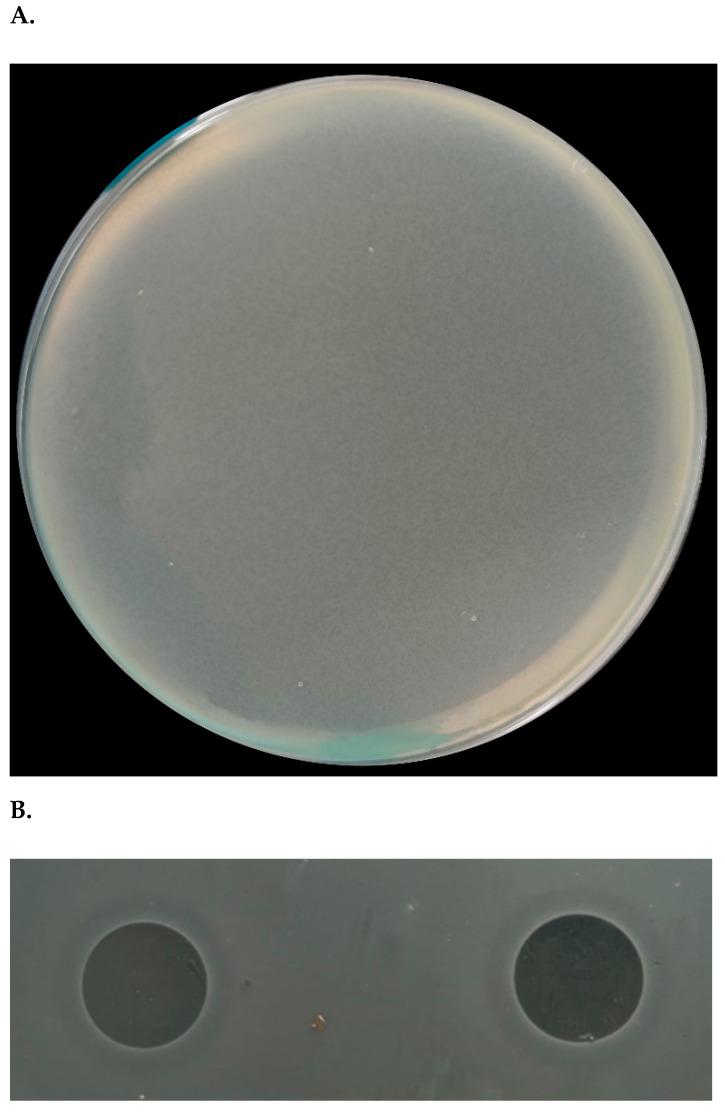

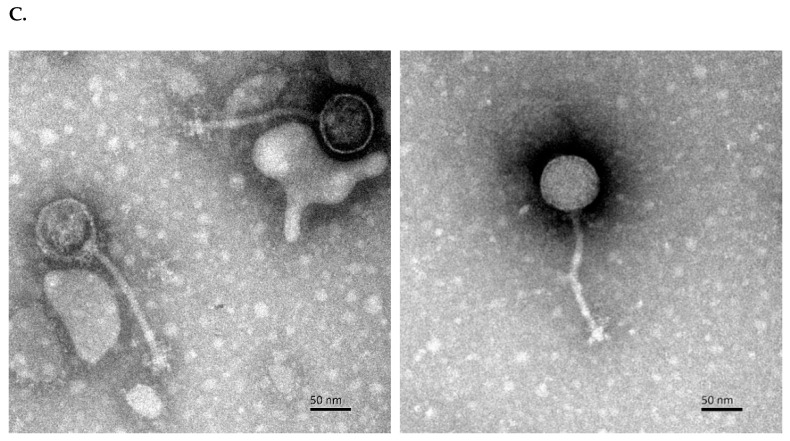
Characterization of the *C. perfringens* phage BG3P. (**A**) Phage lysis plaques on the host (*C. perfringens* BG3) lawn. (**B**) Phage plaque on the host lawn. (**C**) Electron microscopy image of phage BG3P particles. The sample was viewed at a magnification of 200,000×; Scale bar represents 50 nm.

**Figure 2:**
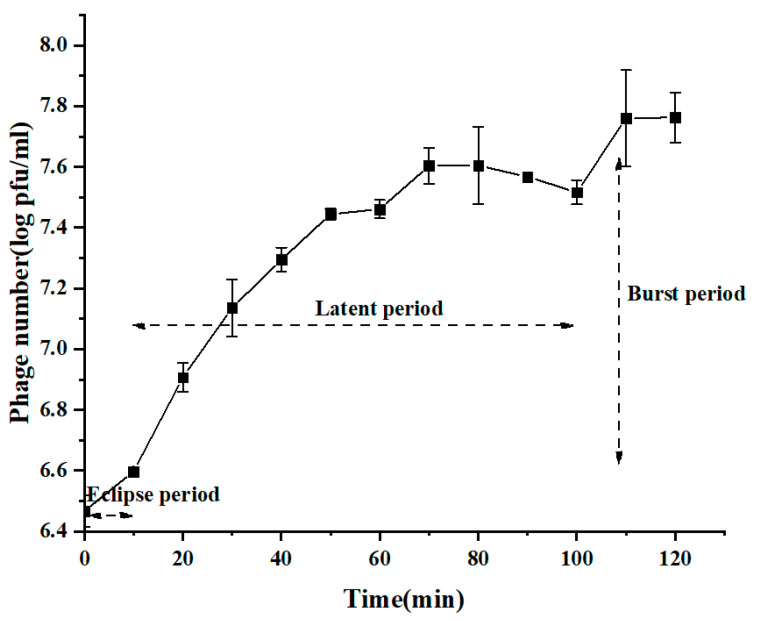
One-step growth curve of phage BG3P infecting its host strain (*C. perfringens* BG3) at 37 °C. Latent and explosion periods are indicated. Values are the mean of three determinations.

In this context, several *C. perfringens* strains were used to isolate specific phages from farm sewage. After enrichment and plaquing, only strain *C. perfringens* BG3 showed phage plaques. This phage, vB_CpeS_BG3P (designated as BG3P hereafter), was able to form clear lysis plaques on the host lawn, with a diameter of about 0.1 mm (Figure 1A). Morphological observation of particles under electronic microscopy showed that phage BG3P had an isometric polyhedral head (70.20 ± 1.25 nm in diameter) and long non-contractile tail (169.64 ± 1.84 nm) (Figure 1B). According to the International Committee on Taxonomy of Viruses (ICTV), phage BG3P was classified into the *Siphoviridae* family. Further characterization of the phage was obtained from the one-step growth curve using a MOI of 10 to infect *C. perfringens* BG3 cultures at 37 °C. Results showed a latent period of about 10 min and an explosion period of 70 min. The average burst size of phages for infected cells was about 18.7 PFU/infected cell (Figure 2).

### 3.2. Phage BG3P Has a Broad Host Range

To determine the host range of phage BG3P, *C. perfringens* strains and other Gram-positive (*Staphylococcus aureus* and *Listeria monocytogenes*) and Gram-negative (*E. coli* and *Salmonella* Enteritidis) bacteria were tested using the spot assay. In addition to four collection strains, 32 *C. perfringens* strains from toxin type A were used. Four toxins were amplified by PCR of 32 strains of *C. perfringens*, and the results showed that only α toxins were amplified positive, so all 32 *C. perfringens* strains were type A *C. perfringens* (Table 1). Phage BG3P exhibited a broad host range against *C. perfringens* isolates: 90.63% (29/32) isolates were inhibited by the phage to varying degrees (Table 1). No lytic activity was detected against other tested bacteria, suggesting that this phage has a species-specific host range spectrum. 

### 3.3. The Stability of Phage BG3P in Response to Temperature and pH Is Suitable for Further Application

**Figure 3:**
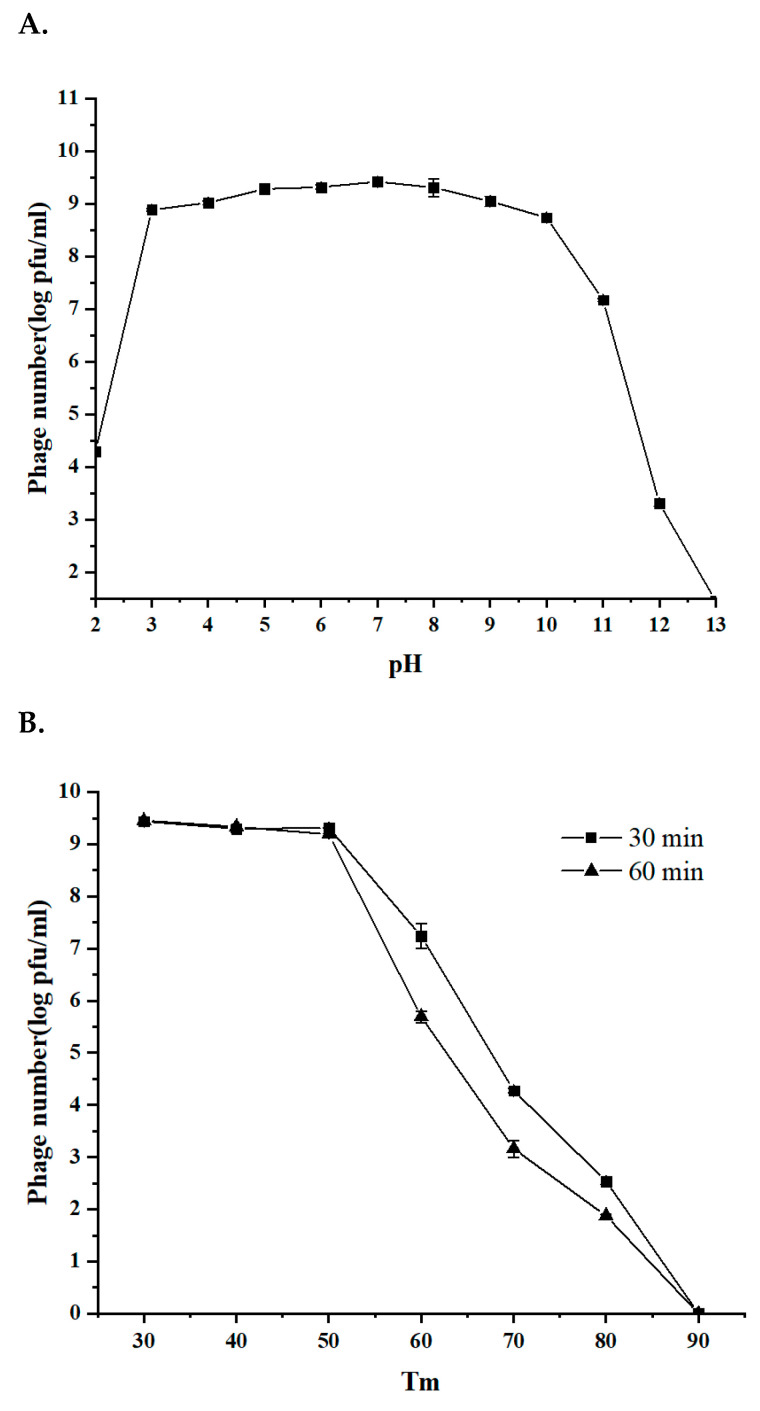
Biological properties of phage BG3P. (**A**) Temperature tolerance in a range from 30 °C to 80 °C. (**B**) pH tolerance after 30 and 60 min of incubation at pH ranging from 2 to 13.Values are the mean of three determinations.

The exploitation of the phage as an antimicrobial requires that the host lysis activity be stable under stress conditions, including high temperature and low pH conditions. Thus, after incubation at 30 °C, 40 °C, and 50 °C, no significant change in the phage number was observed (Figure 3A). However, after incubation at 60 °C for 30 min and 60 min, the phage titer decreased by 2.28 log and 3.50 log, respectively (Figure 3A). A remarkable decrease in phage titer after incubation at 70 °C was observed (5.04 units log and 6.02 units log after 30 min and 60 min, respectively). Similarly, the phage titer fell below 2 log (*p* < 0.01) when treatment was carried out at 80 °C. These results suggest that phage BG3P is thermally stable at a temperature below 50 °C.

With respect to pH sensitivity, phage BG3P was fairly stable in the range of pH 3 to 11 (Figure 3B). The phage titer decreased 1.88 units log at pH 11, and the titer was reduced sharply above this value. This indicated that phage BG3P is resistant to acidic environments and has an average tolerance to alkaline environments.

### 3.4. Quick Adsorption and Efficient Lytic Activity of BG3P

**Figure 4:**
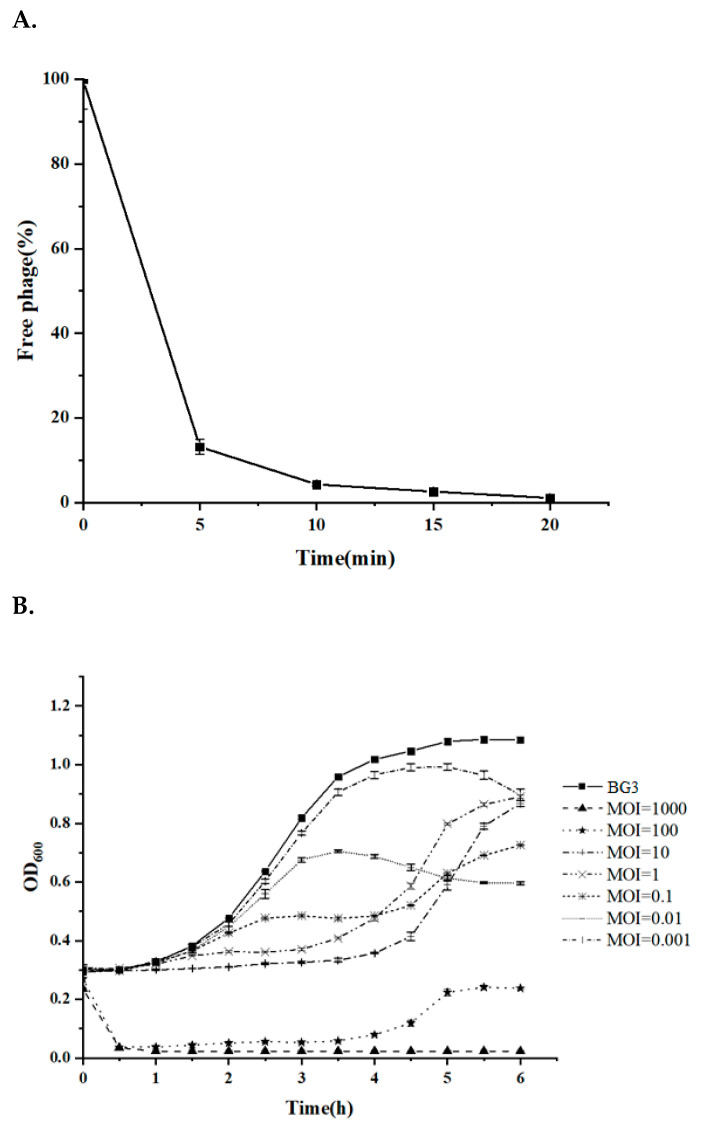

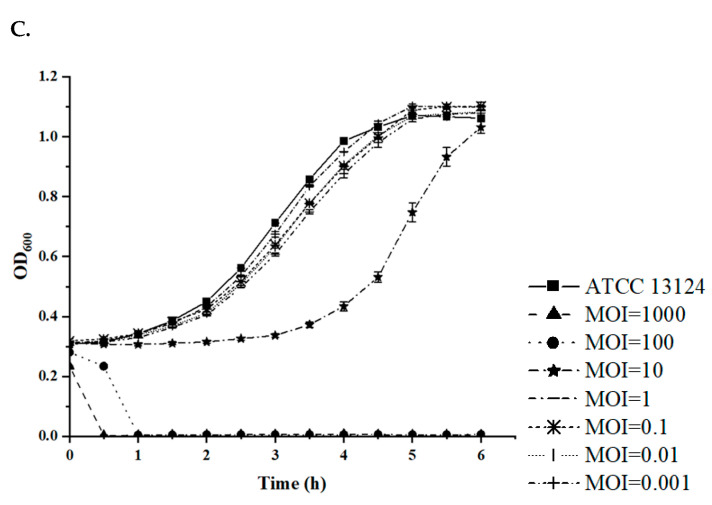
Adsorption and lytic activity of phage BG3P. (**A**) Adsorption of phage BG3P to *C. perfringens* BG3 within 30 min with MOI 0.01. (**B**) Inhibitory effect of phage BG3P against *C. perfringens* BG3 at different MOIs. 1 *×* 10^10^ (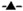), 1 *×* 10^9^ (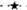), 1 *×* 10^8^ (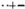), 1 *×* 10^7^ (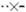), 1 *×* 10^6^ (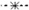), 1 *×* 10^5^ (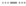), 1 *×* 10^4^ (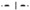) log PFU/mL. The initial concentration of *C. perfringens* BG3 is 10^7^ CFU/mL (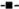). Values are the mean of three determinations. (**C**) Inhibitory effect of phage BG3P against *C. perfringens* ATCC 13,124 at different MOIs. 1 *×* 10^10^ (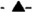), 1 *×* 10^9^ (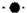), 1 *×* 10^8^ (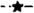), 1 *×* 10^7^ (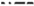), 1 *×* 10^6^ (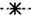), 1 *×* 10^5^ (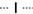), 1 *×* 10^4^ (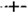) log PFU/mL. The initial concentration of *C. perfringens* ATCC 13,124 is 10^7^ CFU/mL (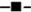). Values are the mean of three determinations.

Adsorption of the phage to the host bacterium is the first step of its life cycle and is essential for its lytic activity. In this context, we determined the adsorption of phage BG3P to *C. perfringens* by measuring the free PFUs in the medium after incubating with *C. perfringens* BG3 cells. The results showed that the percentage of free phages decreased to 14% at 5 min, and less than 10% at 10 min, indicating that phage BG3P adsorbed to host cells quickly, and the adsorption rate reached more than 90% at about 10 min (Figure 4A).

The in vitro lytic activity of the phage against *C. perfringens* cultures was determined by challenge assays using a range of phage concentrations (MOIs) and determining bacteria removed over time. In addition, two different strains (reference strain ATCC 13124 and clinical isolate BG3) were tested in order to compare the effectiveness of the phage. Results showed that phage BG3P at MOI 100 to 1000 could completely inhibit the growth of *C. perfringens* ATCC 13124 within 6 h (Figure 4C). However, the results for *C. perfringens* BG3 were similar and improved slightly after 5 h (Figure 4B). Thus, although all phage concentrations (MOIs ranging from 0.001 and 1000) had an inhibitory effect on bacteria, the time and degree of action were different. Indeed, when the MOI reached 1000, the host strain was completely lysed within 30 min, and re-growth could not be observed after 6 h of incubation for ATCC 13124. A reduction of the MOI (MOI = 100) resulted in an inhibition of the growth of the bacterial culture until 4.5 h, with a light regrowth for BG3. It seems that the BG3P displays a better lytic effect on *C. perfringens* ATCC 13124. Moreover, when we compared the lytic efficiency with a low concentration of BG3P, the result showed that BG3P presents a weakly lytic ability against *C. perfringens* ATCC 13124 (MOI < 100). It seems that a high concentration of phage BG3P could inhibit *C. perfringens* ATCC 13124 completely. However, when the MOI was 1 or 10, no significant CUF increase could be observed in the first 3 h for *C. perfringens* BG3. Overall, the inhibitory effect of the phage on the host is dose-dependent.

### 3.5. The Sequence Analysis of Phage BG3P Genome Revealed That It Is a New Phage

**Figure 5:**
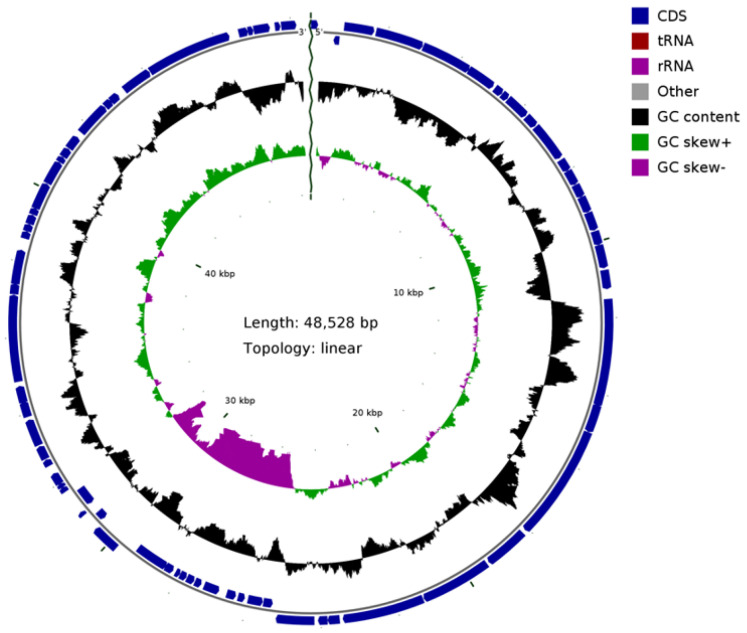
Schematic representation of the phage BG3P genome. Circles display (from the outside): (1) orfs transcribed in the clockwise or the counterclockwise direction. (2) G + C % content. (3) GC skew (G−C/G + C, in a 1-kb window and 0.1-kb incremental shift).

**Figure 6:**
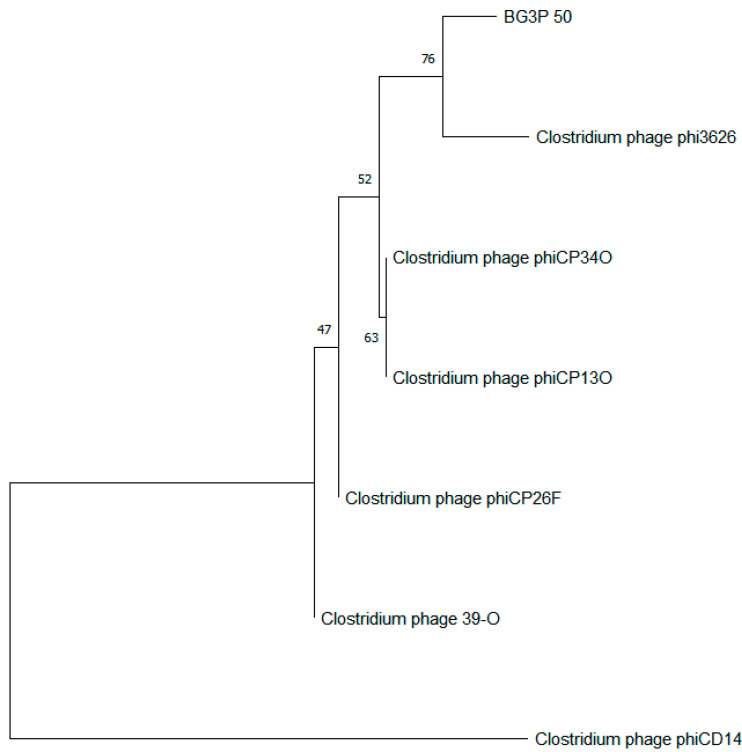
Neighbor-joining phylogenetic trees based on the amino acid sequences of the ssDNA binding protein (*orf 50*) of phage BG3P and other *C. perfringens* phages. The phylogenetic trees were generated using the neighbor-joining method with 1000 bootstrap replicates in MEGA_X_10.2.6. The bar shows the evolutionary distance in the number of amino acid substitutions per site.

The complete genome of phage BG3P is a linear double-stranded DNA with a length of 43,528 bp; it has high identity with the *C. perfringens* phage ΦCP3626 and ΦCPSM101 (Figure 5), with a low GC content of about 28.65% and a 90.33% coding rate. A total of 73 open reading frames (orf) with a minimum size of 123 bp were identified, and the average length of each *orf* was about 600.45 bp (Table 4). Non-coding RNA encoding genes were not predicted in the phage genome. Analysis of the predicted protein sequences by comparing with databases showed some match for 36 proteins, although a putative function only would be ascribed to 13 (17.81%) of them. Orfs were grouped on functional modules (replication, morphogenesis, packaging, and lysis), which were all transcribed on positive strand. Identity percentages were quite low for most of the proteins, ranging 31.4 to 100%. Remarkably, eight orfs showed similarity with phage phiSM101 (identity ranging from 44.7 to 100%), but all of them have unknown function, except the homology to *orf 24* with autolytic activity. This protein was located near to holin and probably is the phage endolysin. Seven orfs were identified as structural protein encoding genes: portal protein (BG3P_05), head morphogenesis protein (BG3P_06), major capsid protein (BG3P_11), tail tape measure protein (BG3P_20), tail protein (BG3P_22), base plate protein (BG3P_24), and neck passage structure protein (BG3P_25). Downstream of the morphogenetic module, proteins identified as belonging to the lysis module were holin (BG3P_27) and lysozyme (BG3P_28), which had similarity with holin from phage phiCT9441A and the autolytic lysozyme encoded by *Clostridium* phage phiSM101, respectively. Moreover, as part of the packaging module we annotated orf BG3P_5 as encoding a putative terminase large subunit, because it was similar to *Clostridioides* phage phiSemix9P1 protein (KX905163.1). Portal protein (BG3P_05) and head morphogenesis protein (BG3P_06) were similar to proteins encoded by phiSemix9P1. Belonging to the replication module, nine gene encoding proteins were identified. BG3P_40 and BG3P_42 showed homology with a replication protein and a transcriptional regulator from *Clostridium* phages phiSM101 and phiSemix9P1, respectively. Orfs BG3P_50, BG3P_52, and BG3P_54 were identified as single-stranded DNA binding protein, nucleoside triphosphate hydrolase, and recombinase, respectively, as they were similar to *Clostridium* phage phiCP9O and *Aeribacillus* phage AP45 proteins. In addition, other proteins belonging to the replication module were BG3P_59, a metal dependent hydrolase; BG3P_60, a nuclease; BG3P_64, a DNA cytosine methyl transferase; and BG3P_68, similar to primase from *Lactobacillus* phage EV3. It should be noted that the BG3P_15 encoding sequence has 97.92% homology to *C. perfringens*-specific chloramphenicol resistance gene (WP_111946556.1)**.** We did not identify any genes encoding virulence and integrase proteins, and there was no evidence that the phage can lysogenize *C. perfringens*. To examine the relationship between phage BG3P and other *C. perfringens* phages, phylogenetic trees were generated using MEGA10 with the amino acid sequences of the phage ssDNA binding protein (*orf 50*), among which only *orf 50* had relatively high homology with the currently published data. When the identity was set to 30%, only six *C. perfringens* phage proteins were classified. In the ssDNA binding protein–based tree, phage BG3P formed a separate cluster together with phage phi3626 (NC_003524.1) (Figure 6). These phylogenetic and genome homology analyses indicated that phage BG3P is a new member of the family *Siphoviridae*.

### 3.6. Chloramphenicol Resistance Gene Transfer

**Figure 7:**
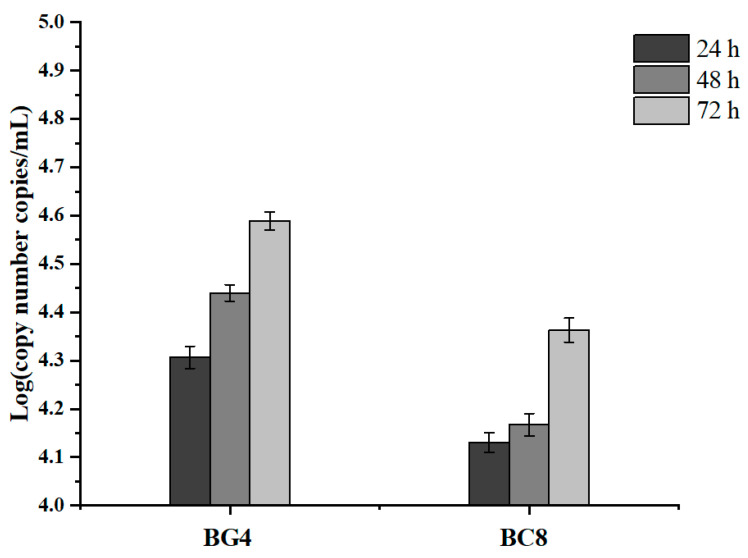
Determination of the *orf 15* gene expression in CM^R^ negative *C. perfringens* strains. Strains BG3 and BG4 not infected by the phage were used as control samples. Control wells always showed negative. Data were analyzed using LightCycler^®^ 480 SW 1.1 software. Mean fold-change values of triplicate independent experiments and standard deviation are shown.

The results showed that 72.72% of the clinical strains carried this Cm^R^ gene, but it was not found in the genome of the host strain BG3 (Table 1). In order to determine whether the putative Cm^R^ gene *orf 15* would be transferred from phage BG3P into the host strain, 10 Cm^R^ negative *C. perfringens* strains (ATCC 13124, CMCC 64722, BG5, RZ-5, BG1, BG3, BC8, BX5, BG4, and BX9-1) were infected with phage BG3P. The results showed that the expression of putative Cm^R^ encoding gene (*orf 15*) could be detected only in two clinical *C. perfringens*, BG4 and BC8, during 72 h incubation, although the CT values were not very high (32.62 and 33.30). According to the standard curve run by phage BG3P DNA standard (y = −3.009X + 41.0779, R^2^ = 0.9995, efficiency of the standard curve is 115%), the expression copy numbers of orf 15 in BG4 and BC8 could be calculated as 3.881 × 10^4^ copies/mL and 2.307 × 10^4^ copies/mL (Figure 7).

Moreover, clones could be seen only in a mixture of *C. perfringens* BG4 and BC8 with phages from the TSC plate without chloramphenicol (10 μg/mL). The other eight strains were lysed after incubating with phage BG3P, and there was no clone growth on the TSC plate. The grown colonies from BG4 and BC8 were picked for anaerobic culture, and the DNA was amplified by PCR with *orf*15 primers. The results showed that *orf 15* gene could be detected in all these colonies, which suggested that the *orf 15* gene could be transferred. We also detected the phage-borne gene *orf 5*, *orf 11*, *orf 25,* and *orf 28* in BG4 and BC8 to confirm if phage BG3P could transfer into its host, and the results showed that *orf 5*, *orf 11*, *orf 25,* and *orf 28* could be amplified from these clones but not the original BG4 and BC8. This may suggest that BG3P has a certain generalized transduction against *C. perfringens,* and there is no transduction phenomenon to other 8 Cm^R^ negative *C. perfringens* strains.

## 4. Discussion

*C. perfringens* has become a significant problem in the poultry industry due to health issues associated with the consumption of contaminated chicken meat, but also due to significant economic losses derived from broilers with low weight gain and increased risk of mortality [27]. Taking into account that a 13% increase in meat production is estimated for the year 2027 (OECD-FAO, 2017), new strategies for controlling this bacterium are mandatory. In this context, *C. perfringens* strains were used to isolate specific phages from farm sewage. A novel *C. perfringens phage* BG3P was isolated and belongs to the *Siphoviridae* family. A few studies have characterized the morphology of *C. perfringens* phages [9,28,29,30,31,32], but they have revealed phages belonging to different families. Other phages belonging to the *Siphoviridae* family are the *C. perfringens* phages ΦSM101, Φ3626, ΦCP39O, ΦCP9O, ΦCP13O, and ΦCP26F [13,33,34]. The phage BG3P was different from *C. perfringens* phages ΦCPV1 and ΦCP24R, which were isolated from broiler chicken intestinal content in Russia and raw sewage from a human waste treatment facility in the U.S. Both *C. perfringens* phages ΦCPV1 and ΦCP24R belonged to the *Podoviridae* family and had an isometric polyhedral head of about 50 nm and a short tail [28,29].

Phage BG3P exhibited a broad host range against *C. perfringens* isolates: 90.63% (29/32) isolates were inhibited by the phage to a varying degree, which was different from the *C. perfringens* phages CPS2 [31] and HN02 [35] reported previously. The host range of phage CPS2 is relatively narrow, phage HN02 showed 42.86% (9/21), and both phages belong to the *Podoviridae* family. With fairly stable tolerance to extremely acidic and alkaline environments, phage BG3P could survive in the gastrointestinal tract of chickens (muscle stomach 2.33~3.52; intestines 6.25~7.21), and therefore, it is feasible to apply it to the biological control of pathogen *C. perfringens*. Moreover, BG3P showed quick adsorption: the adsorption rate reached more than 90% at about 10 min. Conversely, bacteriophage CPS2 took about 40 min to reach more than 90% adsorption, which suggests that phage BG3P displays shorter adsorption time and consequently could have higher efficiency [32].

For the lytic ability, the phage showed different inhibition to the *C. perfringens* strains. *C. perfringens* host strain BG3 was able to completely restrain when infected with high concentration phage BG3P (MOI 1000). In this study, the inhibitory effect of the phage on the host was dose-dependent. Therefore, BG3P has potential for treating antibiotic-resistant *C. perfringens.*

We did not identify any genes encoding virulence and integrase proteins, and there was no evidence that the phage can lysogenize *C. perfringens*. It should be noted that the BG3P_15 encoding sequence had 97.92% homology to *C. perfringens*-specific chloramphenicol resistance protein. A detailed analysis of phage-encoded proteins showed that the protein of *orf 15* is similar to the chloramphenicol resistance protein of *C. perfringens*. Studies have shown that the chloramphenicol resistance gene is carried on movable genetic elements, including the *C. perfringens* transposon Tn 4451 [36] and plasmid pIP401 [37], but it has not been reported on any *C. perfringens* phage. Through the transfer test, *orf 15* was able to transfer to 2 of 10 *C. perfringens* strains, which suggests that phage BG3P could mediate generalized transduction. This has also been reported in previous studies; numerous virulent phages, including Serratia phage ϕIF3 [38], coliphages T1 [39], T4 [40], *Salmonella typhi* phage Vi I [41], *Pseudomonas* phages ϕKZ [42], and *Bacillus* phage SPP1 [43], have been demonstrated to mediate generalized transduction. In addition, some phages are relatively difficult to isolate, such as *Mycobacterium tuberculosis* [44] and *Clostridium difficile* [45]. Similarly, *C. perfringens* phages are also difficult to isolate due to the anaerobic and harsh growth conditions of the host strain. In future, we will further study the relevant mechanisms of *orf 15* gene transfer, so as to further clarify whether it is sporadic or involves other transfer mechanisms.

## 5. Conclusions

*C. perfringens* is a pathogenic bacterium commonly present in soil and in the intestines of humans and animals. In this work, we isolated and characterized a new phage infecting this bacterium and evaluated it as an alternative for biocontrol. Phage BG3P belongs to the family *Siphoviridae,* and its genome sequence had few regions of similarity when compared with the related *C. perfringens* phages in the NCBI databases. In vitro, the lytic effect has the characteristics of wide host range and high lysis efficiency. Remarkably, this phage also has high stability in an alkaline environment. These are interesting properties for the development of intestinal phage applications. In view of the wide host range of phage BG3P and its high lytic activity to *C. perfringens*, in further study, we will consider knocking out the chloramphenicol-like resistance gene and evaluating its application effect and safety, so as to lay a foundation for the development of the *C. perfringens* phage. In addition, we also found that BG3P_27 and BG3P_28 encode holing and autolytic lysozymes. Therefore, we also will try to produce the enzyme in further work.

## Figures and Tables

**Table 1:** Lytic activity and chloramphenicol-resistant gene carrying status of phage BG3P against 39 different strains. For *C. perfringens* strains of poultry origin, the toxin type is indicated.

Strain	Type	BG3P	Source	*orf 15*
BX3	A	**++**	Chicken heart feces	**+**
BG9-1	A (NetB toxin positive)	**+**	Chicken liver feces	**+**
ZA2	A	**++**	Chicken intestinal feces	**+**
BG5	A	**++**	Chicken liver feces	**-**
RZ-5	A (NetB toxin positive)	**++**	Chicken intestinal feces	**-**
BP1	A	**++**	Chicken spleen feces	**+**
BX4	A	**++**	Chicken heart feces	**+**
BC5	A	**+**	Chicken intestinal feces	**+**
BX1	A	**++**	Chicken heart feces	**+**
BC7	A (NetB toxin positive)	**+**	Chicken intestinal feces	**+**
ZA4	A	**+**	Chicken intestinal feces	**+**
BG1	A	**++**	Chicken liver feces	**-**
BG3	A	**++**	Chicken liver feces	**-**
G1-12	A	**-**	Chicken intestinal feces	**+**
BC8	A	**++**	Chicken intestinal feces	**-**
G2-4	A	**+**	Chicken intestinal feces	**+**
BX5	A	**+**	Chicken liver feces	**-**
ZA3	A (NetB toxin positive)	**++**	Chicken intestinal feces	**+**
ZA1	A (NetB toxin positive)	**+**	Chicken intestinal feces	**+**
BC4	A	**+**	Chicken intestinal feces	**+**
BP3	A	**++**	Chicken spleen feces	**+**
BG7	A	**+**	Chicken liver feces	**+**
RZ-1	A	**+**	Chicken intestinal feces	**+**
BG4	A	**++**	Chicken liver feces	**-**
G3-2	A	**+**	Chicken intestinal feces	**+**
BX6	A	**+**	Chicken liver feces	**+**
BX9-1	A (NetB toxin positive)	**+**	Chicken liver feces	**-**
BP5	A	**+**	Chicken spleen feces	**+**
BC3	A	**-**	Chicken intestinal feces	**+**
G2-2	A	**+**	Chicken intestinal feces	**+**
RZ-4	A	**+**	Chicken intestinal feces	**+**
BP9-1	A (NetB toxin positive)	**-**	Chicken spleen feces	**+**
*C. perfringens* ATCC 13124	A	**+**	Our collection or Reference	**-**
*C. perfringens* ATCC 64722	A	**+**		
*C. perfringens* ATCC 64723	A	**+**		
*C. perfringens* ATCC 64724	A	**+**		
*Staphylococcus aureus* ATCC 25923	**-**	**-**	**-**	
*Salmonella enteritidis* ATCC 13076	**-**	**-**	**-**	
*Escherichia coli* ATCC 25922	**-**	**-**	**-**	
*Listeria monocytogenes* ATCC 19114	**-**	**-**	**-**	

“++” transparent, “+” semi-transparent, “-” negative.

**Table 2:** Toxin typing primers of *C. perfringens*.

	Primer Sequence 5′-3′	Size (bp)
α (cpa)-F	GCTAATGTTACTGCCGTTGA	324
α (cpa)-R	CCTCTGATACATCGTGTAAG	
β (cpb)-F	GCGAATATGCTGAATCATCTA	196
β (cpb)-R	GCAGGAACATTAGTATATCTTC	
ε (etx)-F	GCGGTGATATCCATCTATTC	655
ε (etx)-R	CCACTTACTTGTCCTACTAAC	
ι (iap)-F	ACTACTCTCAGACAAGACAG	446
ι (iap)-R	CTTTCCTTCTATTACTATACG	

**Table 3:** Nucleotide sequence of primers used for PCR amplification of putative portal protein gene *orf 5*, putative major capsid protein gene *orf 11*, putative chloramphenicol resistant gene *orf 15*, putative neck passage structure protein gene *orf 25*, putative autolytic lysozyme gene *orf 28*.

Gene	Sequence 5′-3′	Amplicon Size (bp)
*orf 5*	CGACCGAAGGATACGCATT	736
	GCAAGAATTTGGGCAGTCATTA	
*orf 11*	TTACTGGAGCGAACGGACTT	776
	ACCAACCATCTTAGCTTCTTCC	
*orf 15*	GGGAATTCCATATGAAAAAGTGTCCGCATTTAGAA	435
	CCGCTCGAGCTGCGCTTTATCAATATCTGT	
*orf 25*	TACTAATGCTGCTGAAGGTGAA	1924
	TGCAAGCGGATTGCTTAACA	
*orf 28*	AATGCTAAGAGCTGGATATGGT	862
	GCCAATAAAGTCTGAGTCTACC	

**Table 4:** Functional grouping of predicted orfs in phage BG3P and their similarity with databases.

Gene ID	Sbjct ID	Organism	Hit_Description	Identity(%)	*E* Value
BG3P_1				None	
BG3P_2				None	
BG3P_3	YP_001426191.1	*Chlorella* virus FR483	hypothetical protein FR483_N559R	32.23	2.50E-10
BG3P_4	YP_007237242.1	*Clostridium* phage phi8074-B1	putative terminase large subunit	52.00	3.00E-53
BG3P_5	ARB07045.1	*Clostridioides* phage phiSemix9P1	portal protein	47.79	4.10E-98
BG3P_6	ARB07046.1	*Clostridioides* phage phiSemix9P1	head morphogenesis protein	31.47	1.30E-19
BG3P_7				None	
BG3P_8				None	
BG3P_9	ARB07047.1	*Clostridioides* phage phiSemix9P1	hypothetical protein Semix9P1_phi04	33.00	9.60E-21
BG3P_10				None	
BG3P_11	YP_008858642.1	*Brevibacillus* phage Davies	major capsid protein	45.35	4.30E-71
BG3P_12				None	
BG3P_13	YP_009214137.1	*Clostridium* phage phiCD146	conserved protein of unknown function	36.30	5.80E-19
BG3P_14				None	
BG3P_15	WP_111946556.1	*Clostridium perfringens* strain CPI 103K-3 plasmid pCPCPI103K-3_3	chloramphenicol resistance protein	97.92	1E-99
BG3P_16				None	
BG3P_17				None	
BG3P_18				None	
BG3P_19	YP_009215039.1	*Brevibacillus* phage Osiris	hypothetical protein OSIRIS_25	36.36	1.30E-07
BG3P_20	YP_006383517.1	*Clostridium* phage phiS63	*Clostridium* phage phiS63 gp13	35.66	4.00E-95
BG3P_21				None	
BG3P_22	YP_006383519.1	*Clostridium* phage phiS63	*Clostridium* phage phiS63 gp15	53.85	7.00E-16
BG3P_23	ARQ95837.1	*Staphylococcus* phage qdsa001	hypothetical protein qdsa001_81	30.99	6.50E-23
BG3P_24	YP_006383520.1	*Clostridium* phage phiS63	*Clostridium* phage phiS63 gp16	83.25	0.00E+00
BG3P_25	YP_009188471.1	*Streptococcus* phage Str-PAP-1	neck passage structure protein	31.93	4.40E-41
BG3P_26				None	
BG3P_27	YP_009219438.1	*Clostridium* phage phiCT9441A	holin	53.09	1.30E-16
BG3P_28	YP_699978.1	*Clostridium* phage phiSM101	autolytic lysozyme	48.26	5.70E-76
BG3P_29	YP_699977.1	*Clostridium* phage phiSM101	hypothetical protein CPR_C0049	95.35	1.80E-37
BG3P_30	YP_699976.1	*Clostridium* phage phiSM101	hypothetical protein CPR_C0048	97.08	6.20E-69
BG3P_31	YP_699975.1	*Clostridium* phage phiSM101	hypothetical protein CPR_C0047	100.00	2.70E-27
BG3P_32	YP_699974.1	*Clostridium* phage phiSM101	hypothetical protein CPR_C0046	92.94	9.30E-39
BG3P_33	YP_699973.1	*Clostridium* phage phiSM101	hypothetical protein CPR_C0045	94.12	2.20E-61
BG3P_34	YP_699972.1	*Clostridium* phage phiSM101	hypothetical protein CPR_C0044	100.00	7.50E-30
BG3P_35				None	
BG3P_36				None	
BG3P_37				None	
BG3P_38				None	
BG3P_39				None	
BG3P_40	YP_699968.1	*Clostridium* phage phiSM101	hypothetical protein CPR_C0040	44.79	2.50E-41
BG3P_41				None	
BG3P_42				None	
BG3P_43	ARB07097.1	*Clostridioides* phage phiSemix9P1	y4mF family transcriptional regulator	42.37	7.90E-18
BG3P_44				None	
BG3P_45				None	
BG3P_46				None	
BG3P_47				None	
BG3P_48	YP_006990357.1	*Streptococcus* phage phiNJ2	hypothetical protein phiNJ2_0038	41.41	1.60E-12
BG3P_49	APC46471.1	*Aeribacillus* phage AP45	hypothetical protein	49.33	4.50E-51
BG3P_50	YP_002265460.1	*Clostridium* phage phiCP9O	single-stranded DNA-binding protein	59.56	4.10E-33
BG3P_51				None	
BG3P_52	APC46462.1	*Aeribacillus* phage AP45	nucleoside triphosphate hydrolase	38.79	
BG3P_53				None	
BG3P_54	APC46463.1	*Aeribacillus* phage AP45	recombinase	44.44	1.80E-60
BG3P_55				None	
BG3P_56				None	
BG3P_57				None	
BG3P_58	YP_009223784.1	*Geobacillus* virus E3	hypothetical protein E3_077	50.94	3.50E-13
BG3P_59	YP_009151501.1	*Bacillus* phage Pascal	Metal-dependent hydrolase	44.26	9.60E-46
BG3P_60	YP_002265457.1	*Clostridium* phage phiCP39-O *Clostridium* phage phiCP13-O	hypothetical protein 39-O_gp49 hypothetical protein 13-O_gp46	41.87 58.1	1.60E-39
BG3P_61				None	
BG3P_62				None	
BG3P_63				None	
BG3P_64	AFB75778.1	unidentified phage	hypothetical protein 2019_scaffold132_00014	65.40	1.10E-07
BG3P_65	YP_006907549.1	*Bacillus* phage Bastille	hypothetical protein	43.66	1.80E-08
BG3P_66				None	
BG3P_67	Q9PD82			37.93	3.00E-12
BG3P_68	CUQ99459.1	*Lactobacillus* phage EV3	phage primase	36.57	4.00E-77
BG3P_69				None	
BG3P_70				None	
BG3P_71				None	
BG3P_72				None	
BG3P_73	YP_007005016.1	*Clostridium* phage phiCP13O	hypothetical protein phi13O_gp1	44.26	8.70E-20

## Data Availability

The data presented in this study are available on request from the corresponding author.
